# Hypertension, cerebral Amyloid, aGe Associated Known neuroimaging markers of cerebral small vessel disease Undertaken with stroke REgistry (HAGAKURE) prospective cohort study: Baseline characteristics and association of cerebral small vessel disease with prognosis in an ischemic stroke cohort

**DOI:** 10.3389/fnagi.2023.1117851

**Published:** 2023-03-02

**Authors:** Shuhei Ikeda, Yusuke Yakushiji, Jun Tanaka, Masashi Nishihara, Atsushi Ogata, Makoto Eriguchi, Shohei Ono, Masafumi Kosugi, Kohei Suzuyama, Megumi Mizoguchi, Chika Shichijo, Toshihiro Ide, Yukiko Nagaishi, Hodo Mori, Natsuki Ono, Masaaki Yoshikawa, Kiku Ide, Hiromu Minagawa, Kotaro Iida, Kazuhiro Kawamoto, Yoshiko Katsuki, Hiroyuki Irie, Tatsuya Abe, Hideo Hara

**Affiliations:** ^1^Division of Neurology, Department of Internal Medicine, Faculty of Medicine, Saga University, Saga, Japan; ^2^Department of Neurology, Kansai Medical University, Hirakata, Japan; ^3^Department of Radiology, Faculty of Medicine, Saga University, Saga, Japan; ^4^Department of Neurosurgery, Faculty of Medicine, Saga University, Saga, Japan

**Keywords:** small vessel disease (SVD), cerebral Amyloid aGe (CAA), magnetic resonance imaging (MRI), hypertension, ischemic stroke (IS)

## Abstract

**Introduction:**

Cerebral small vessel disease (SVD) is one of the leading causes of stroke; each neuroimaging marker of SVD is correlated with vascular risk factors and associated with poor prognosis after stroke. However, longitudinal studies investigating the association between comprehensive SVD burden scoring system, “total SVD score” – which encompasses the established neuroimaging markers of lacunae, cerebral microbleeds (CMBs), white matter hyperintensities (WMH) including periventricular hyperintensities, and perivascular spaces in basal ganglia– and clinical outcomes are limited. The aim of this study is to determine the association between SVD burden and long-term prognosis in patients with ischemic stroke.

**Methods and design:**

This prospective, single-center, observational study enrolled patients with acute ischemic stroke, including cerebral infarction and transient ischemic attack. Magnetic resonance imaging scans were performed, and then total SVD score (range, 0–4) was calculated. We recorded baseline characteristics and evaluated the relationships of long-term outcomes to SVD neuroimaging markers and total SVD score. Stroke recurrence was thought as primary outcome. Hazard ratios (HRs) of events during follow-up were calculated using Cox proportional hazards modeling with adjustments for age, sex, hypertension, dyslipidemia, diabetes mellitus, atrial fibrillation, and smoking. Cumulative event rates were estimated using the Kaplan–Meier method.

**Results:**

Consecutive 564 acute ischemic stroke patients were enrolled according to inclusion and exclusion criteria. A total of 467 participants with first-ever ischemic stroke were analyzed (median age 75.0 [interquartile range, 64.0–83.0] years, 59.3% male). Total SVD score was 0 point in 47 individuals (12.0%), 1 point in 83 (21.2%), 2 points in 103 (26.3%), 3 points in 85 (21.7%), and 4 points in 73 (18.7%). Twenty-eight recurrent stroke events were identified during follow-up. Total SVD score ≥ 2, presence of CMBs, and moderate-to-severe WMH were associated with increased risk of recurrent stroke events (HR 9.31, 95% confidence interval [CI] 2.33–64.23; HR 2.81, 95% CI 1.08–7.30; HR 2.90, 95% CI 1.22–6.88, respectively).

**Conclusion:**

The accumulation of SVD biomarkers as determined by total SVD score offered a reliable predictor of stroke recurrence. This study established a firm understanding of SVD prognosis in clinical settings.

## Introduction

Cerebral small vessel disease (SVD) refers to various pathological, clinical, and neuroimaging changes caused by damage to the perforating cerebral arterioles, capillaries, and venules. Neuroimaging markers of SVD are commonly seen in stroke patients and are considered to be associated with poor prognosis after stroke, including post-stroke dementia, recurrent stroke, and mortality (Greenberg et al., 2004; Best et al., 2021; Pasi et al., 2021). Effective treatment and prevention of stroke recurrence requires a better understanding of the mechanisms and underlying spectrum of SVD. Among the proposed scoring systems for comprehensive SVD burden, “total SVD score” – which encompasses the four established neuroimaging markers of lacunae, cerebral microbleeds (CMBs), white matter hyperintensities (WMH) including periventricular hyperintensities (PVH), and perivascular spaces in basal ganglia (BG-PVS) – has consistently displayed strong associations with traditional vascular risk factors.

However, longitudinal studies systematically investigating associations between scoring and clinical outcomes have been limited (Lau et al., 2017). We therefore initiated a prospective observational study named HAGAKURE (Hypertension, cerebral Amyloid, aGe Associated Known neuroimaging markers of cerebral small vessel disease Undertaken with stroke REgistry) to explore longitudinal associations between SVD and prognosis with recurrent stroke using a stroke cohort. In this article, we detail the protocol for the ischemic stroke (IS) cohort of the HAGAKURE study. Moreover, we present baseline data and associations of neuroimaging markers for SVD with long-term prognosis among patients with IS.

## Materials and methods

### Study design

The HAGAKURE study is a Japanese prospective single-center hospital-based observational study investigating the prognosis of SVD at 3, 12, and 24 months after stroke onset. The study is being performed and reported with reference to the Strengthening the Reporting of Observational Studies in Epidemiology guidelines (von Elm et al., 2007).

### Ethics

The study protocol was approved by the ethics committee at our hospital (approval nos. 24-26, 27-45, and 29-55) and is registered with the University Hospital Medical Information Network clinical trial registry in Japan (UMIN 000037894). The investigators obtained written informed consent from patients or their family members before registration. The study is being performed in accordance with the principles of the Declaration of Helsinki 59th WMA General Assembly, Seoul, (October 2008). SI and YY had full access to all the data in the study and take responsibility for the integrity of the data and the accuracy of the data analysis. Our dataset can be shared on reasonable request after appropriate approval.

### Participants

**FIGURE 1:**
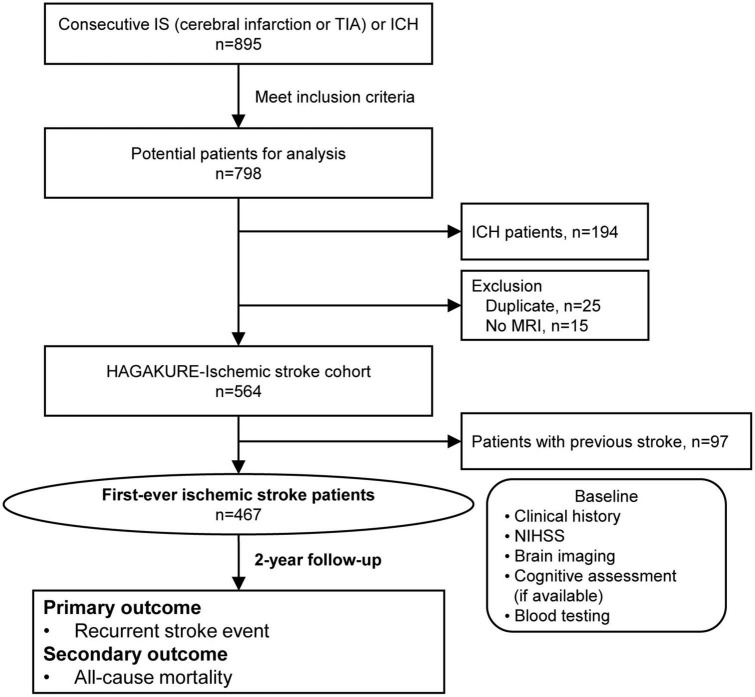
Flow diagram of patient selection for the HAGAKURE study. The HAGAKURE study is a single-center, prospective, longitudinal, observational study investigating the association between cerebral small vessel disease burden in patients with stroke (cerebral infarction, TIA, or ICH) and their associations (e.g., outcomes). ICH, intracerebral hemorrhage; IS, ischemic stroke; MRI, magnetic resonance imaging; TIA, transient ischemic attack.

Table 1 shows the inclusion and exclusion criteria for the IS cohort of this study. Patients with IS (i.e., cerebral infarction or transient ischemic attack [TIA]) admitted to our division within a week after onset were considered eligible for the study. Exclusion criteria were: (1) duplicated registration; or (2) unavailability of brain magnetic resonance imaging (MRI) data. Eligible individuals are shown in Figure 1.

**TABLE 1:** Eligibility criteria for patient registration in the ischemic stroke cohort.

Inclusion criteria
• Ischemic stroke (cerebral infarction or TIA) ≤ 7 days from symptom onset
• Admission for ischemic stroke between September 2012 and August 2016
• Age ≥ 20 years at onset of stroke
• Provision of written informed consent either directly or by suitable surrogate
**Exclusion criteria**
• Duplicate registration
• Brain magnetic resonance imaging unavailable

TIA, transient ischemic attack.

### Baseline assessments

Using a standardized case record form, data on demographics, medical history, medication use, physical examination, lifestyle, and pre-stroke functional status were collected. The details of these variables are described in the supplemental methods. We did not obtain ethnicity data directly from the patient or their family. Instead, we retrospectively checked the medical records (place of birth, name, family structure, facial photograph), and judged that all patients were East Asian. At baseline, we recorded stroke severity using the National Institutes of Health Stroke Scale score. These data were entered into the database by a single research nurse (YK) and double-checked by the co-authors (YY, JT, ME, MK, KS, MM, CS, YN, or NO).

### Definition and classification of IS

The IS cohort comprised patients with cerebral infarction or TIA. Cerebral infarction was defined as sudden neurological dysfunction with evidence of acute infarction on brain imaging, regardless of symptom duration. TIA was defined as a transient episode of neurological dysfunction caused by focal brain ischemia without evidence of acute infarction on brain imaging (Easton et al., 2009). Subtypes of IS were classified according to the classification of Trial of Org 10172 in Acute Stroke Treatment (Adams et al., 1993).

### Imaging data collection

All patients were evaluated by brain MRI at baseline. Sequences of axial brain MRI and the associated parameters are described in Supplementary Table 1. Digital Imaging and Communications in Medicine (DICOM)^®^ data from MRI for use in rating were selected according to priori-defined criteria as described in Supplementary Table 2. DICOM data were anonymized and uploaded by a single co-author (S.O.) to a central imaging lab, and viewed on OsiriX medical imaging software (OsiriX MD; Pixmeo, Geneva, Switzerland) using a dedicated monitor (EIZO MX270W; EIZO Corporation, Ishikawa, Japan).

### Definition and classification of neuroimaging markers and total SVD scores

The definitions and classifications of neuroimaging markers, including lacunae (of presumed vascular origin), CMBs, WMH (of presumed vascular origin), and BG-PVS were classified according to recent consensus criteria and validated scales (Pasquier et al., 1996; Gregoire et al., 2009; Wardlaw et al., 2013; Charidimou et al., 2015), as described in Supplementary Table 3. Our main interest in this study was in identifying the total SVD score. According to a recently developed scoring system (Staals et al., 2014), total SVD scores were assigned as follows: presence of lacunae or CMBs was defined as the presence of one or more foci (1 point each if present); moderate-to-severe WMH including PVH was defined as either (early) confluent deep WMH (Fazekas score 2 or 3) and/or irregular PVH extending into deep white matter (Fazekas score 3) (1 point if present); and presence of moderate-to-severe BG-PVS (i.e., ≥ 11 BG-PVS on one side) (1 point if present). Total SVD score thus ranged from 0 to 4.

### Rating of neuroimaging markers

Neuroimaging markers on MRI were assessed by three raters, comprising a single board-certified neuroradiologist (MN) and two neurologists (YY and JT). All raters were blinded to all clinical information. Inter-rater reliability for these findings was calculated by comparing the certified neuroradiologist and neurologists using 40 MRI scans that were randomly selected. Intra-rater reliability for these tests was scored twice with an interval of ≥ 4 weeks, with raters blinded to the initial ratings.

### Other evaluations at baseline

Other evaluations and methodologies at baseline (i.e., cognitive function, blood tests, and physiological and imaging evaluations) are described in the supplemental methods.

### Outcomes and follow-up strategy

The primary outcome was defined as recurrent stroke (i.e., cerebral infarction, TIA, intracerebral hemorrhage [ICH], or subarachnoid hemorrhage treated with admission). The secondary outcome was defined as all-cause mortality. To obtain information about the events, we sent periodic (3, 12, and 24 months after stroke onet) questionnaires to all participants (or conducted telephone surveys for patients who did not reply). When cerebrovascular events were reported, the authors checked the medical records and images of brain computed tomography and/or MRI. If respondents were lost to follow-up, data obtained from the latest questionnaire were used as the final records. Participants with incomplete information for follow-up questionnaires or with follow-up duration < 3 month were excluded. The HAGAKURE study commenced in September 2012 and final follow-up finished in March 2019.

### Sample size calculation

In a previous longitudinal follow-up study in Japan (Hata et al., 2005), 10% of participants experienced a subsequent cerebrovascular event within 2 years of follow-up. Based on this, to perform multivariate analysis with about six variables, 60 participants with recurrent stroke within the first 2 years after stroke were required. We aimed to recruit up to 600 IS patients and 200 ICH patients, to allow for 25% loss to follow-up.

### Statistical analysis

We analyzed associations between baseline characteristics and recurrent stroke events or all-cause mortality using Student’s t test for continuous variables and the χ^2^ test for categorical variables. These outcomes were evaluated using Kaplan–Meier curves. We determined both unadjusted and adjusted (for age, sex, and vascular risk factors) risks of recurrent stroke and all-cause mortality in patients with: (1) total SVD score ≥ 3; (2) total SVD score ≥ 2; and (3) increasing burden of each neuroimaging marker through Cox regression analysis. Covariates including MRI results and clinical biomarkers were selected according to previous studies. Two-sided testing was performed with a significance level of 5%. The 95% confidence intervals (CI) were two-sided. All analyses were performed using SPSS version 23.0 software (IBM Corporation, Armonk, NY) and JMP version 15.0 software (SAS Institute, Cary, NC).

### Study organization and funding

The HAGAKURE was organized by a central coordinating center located at Saga University Hospital and an associated center at Kansai Medical University, with funding support by a Grant-in-Aid for Scientific Research (C), JSPS KAKENHI (grant numbers 15K10364 and 21K10510).

## Results

A flow diagram of patient selection for the IS cohort of the HAGAKURE study is shown in Figure 1. A total of 895 consecutive patients were admitted to our division with acute cerebral infarction, TIA, and ICH. Of those, 604 patients met the inclusion criteria for the IS cohort, then another 40 patients were excluded: 25 due to duplications; 15 due to lack of MRI data. A final total of 564 patients were included in this study, then 467 patients were diagnosed with first-ever IS. Baseline characteristics and imaging findings are shown in Table 2. The most common subtype of IS was cardioembolism (31.1%), followed by undetermined cause (21.6%), large-artery atherosclerosis (21.2%), small vessel occlusion (15.6%), and other cause (10.5%). Median duration from admission to brain MRI for the evaluation of SVD markers was 8 days (interquartile range [IQR] 5–10 days). Inter- and intra-rater reliabilities of SVD markers on MRI are shown in Supplementary Table 4. Proportions of each total SVD score were as follows: 0 points, 12.0%; 1 point, 21.2%; 2 points, 26.3%; 3 points, 21.7%; and 4 points, 18.7%. Main baseline laboratory data are shown in Supplementary Table 5. During the study periods, 36 patients were excluded from the analysis of recurrent stroke events, and 8 patients were excluded from the analysis of all-cause mortality due to inadequate duration of follow-up. A total of 28 recurrent stroke events (mean follow-up, 27 ± 12 months) and 104 cases of all-cause mortality (mean follow-up, 26 ± 13 months) occurred. Univariate analyses for prognosis are shown in Table 3.

**TABLE 2:** Baseline clinical characteristics in first-ever ischemic stroke patients.

	First-ever ischemic stroke patients (*n* = 467)
Age, years	75.0 [64.0–83.0]
Sex, male	277 (59.3)
Body mass index, kg/m^2^	22.7 ± 3.8
Systolic blood pressure, mmHg	159.9 ± 31.2
Diastolic blood pressure, mmHg	88.1 ± 20.7
Modified Rankin Scale score	0 [0–0]
**Risk factors**	
Hypertension	347 (74.3)
Diabetes mellitus	155 (33.7)
Dyslipidemia	287 (62.5)
Current smoking	108 (23.2)
**Comorbidities**	
Atrial fibrillation	145 (31.1)
Previous ischemic heart disease	41 (8.8)
NIHSS score	4.0 [2.0–14.0]
Antithrombotic agents	122 (26.1)
**Stroke subtype**	
Small vessel occlusion	73 (15.6)
Cardioembolism	145 (31.1)
Large-artery atherosclerosis	99 (21.2)
Other	49 (10.5)
Undetermined	101 (21.6)
**Neuroimaging marker**	
Lacunae	173 (37.3)
CMBs	176 (38.1)
Moderate-to-severe WMH	252 (54.1)
Moderate-to-severe BG-PVS	334 (85.0)
**Total SVD score**	
0	47 (12.0)
1	83 (21.2)
2	103 (26.3)
3	85 (21.7)
4	73 (18.7)

Body mass index data are missing for 10 patients, diabetes mellitus for 7, dyslipidemia for 8, and current smoking for 1. Total SVD score were evaluated for 391 patients. BG-PVS, perivascular spaces in basal ganglia; CMBs, cerebral microbleeds; NIHSS, National Institutes of Health Stroke Scale; SVD, small vessel disease; WMH, white matter hyperintensities.

**TABLE 3:** Univariate analysis for prognosis in first-ever ischemic stroke patients.

	Recurrent stroke			All-cause mortality		
	Event (–) (*n* = 403)	Event (+) (*n* = 28)	*p*-value	Event (–) (*n* = 355)	Event (+) (*n* = 104)	*p*-value
Age, years	75.0 [63.0–83.0]	74.5 [66.0–83.8]	0.641	72.0 [61.0–81.0]	83.0 [76.0–88.0]	< 0.001
Sex, male	236 (58.6)	21 (75.0)	0.087	212 (59.7)	59 (56.7)	0.586
Body mass index, kg/m^2^	22.8 ± 3.8	23.3 ± 2.7	0.390	23.3 ± 3.6	20.7 ± 3.7	< 0.001
Systolic blood pressure, mmHg	160.3 ± 31.1	157.4 ± 25.0	0.682	161.2 ± 31.0	155.3 ± 31.2	0.301
Diastolic blood pressure, mmHg	88.3 ± 20.6	85.0 ± 16.3	0.382	89.2 ± 20.1	83.9 ± 22.2	0.036
Modified Rankin Scale score at baseline	0 [0.0–0.0]	0 [0.0–0.0]	0.733	0 [0.0–0.0]	0.5 [0.0–2.0]	< 0.001
**Risk factors**						
Hypertension	303 (75.2)	22 (78.6)	0.688	270 (76.1)	73 (70.2)	0.226
Diabetes mellitus	137 (34.5)	4 (14.3)	0.028	116 (33.1)	37 (36.6)	0.502
Dyslipidemia	251 (63.2)	20 (74.1)	0.256	231 (65.8)	51 (51.0)	0.007
Current smoking	97 (24.1)	7 (25.0)	0.917	96 (27.1)	10 (9.6)	< 0.001
**Comorbidities**						
Atrial fibrillation	118 (29.3)	8 (28.6)	0.936	85 (23.9)	58 (55.8)	< 0.001
Previous ischemic heart disease	35 (8.7)	2 (7.1)	0.778	34 (9.6)	6 (5.8)	0.226
NIHSS score	4.0 [2.0–13.0]	2.0 [2.0–4.8]	0.054	3.0 [1.0–8.0]	15.5 [3.3–25.0]	< 0.001
Antithrombotic agents	100 (24.8)	11 (39.3)	0.090	88 (24.8)	33 (31.7)	0.158
Stroke subtype			0.398			0.006
Small vessel occlusion	66 (16.4)	4 (14.3)		59 (16.6)	12 (11.5)	
Cardioembolism	122 (30.3)	5 (17.9)		95 (26.8)	48 (46.2)	
Large-artery atherosclerosis	88 (21.8)	10 (35.7)		83 (23.4)	16 (15.4)	
Other	41 (10.2)	2 (7.1)		37 (10.4)	9 (8.7)	
Undetermined	86 (21.3)	7 (25.0)		81 (22.8)	19 (18.3)	
**Neuroimaging markers**						
Lacunae	144 (35.8)	14 (51.9)	0.095	125 (35.4)	42 (40.8)	0.320
CMBs	142 (35.4)	16 (59.3)	0.013	129 (36.7)	41 (40.2)	0.514
Moderate-to-severe WMH	216 (53.6)	20 (71.4)	0.067	183 (51.6)	65 (63.1)	0.038
Moderate-to-severe BG-PVS	293 (84.0)	21 (91.3)	0.347	254 (81.7)	73 (97.3)	< 0.001
Total SVD score			0.002			0.033
0	44 (12.7)	2 (8.7)		45 (14.6)	2 (2.7)	
1	81 (23.3)	0		69 (22.3)	14 (18.7)	
2	87 (25.1)	9 (39.1)		77 (24.9)	25 (33.3)	
3	76 (21.9)	2 (8.7)		65 (21.0)	16 (21.3)	
4	59 (17.0)	10 (43.5)		53 (17.2)	18 (24.0)	

A total of 36 patients with recurrent stroke events and 8 patients with all-cause mortality were excluded from analysis because the follow-up period was less than 90 days. Data are presented as n (%), mean ± standard deviation, or median [interquartile range]. Data on body mass index are missing for 7 patients, diabetes mellitus for 6, dyslipidemia for 7, and current smoking for 1. BG-PVS, perivascular spaces in basal ganglia; CMBs, cerebral microbleeds; NIHSS, National Institutes of Health Stroke Scale; SVD, small vessel disease; WMH, white matter hyperintensities.

**FIGURE 2:**
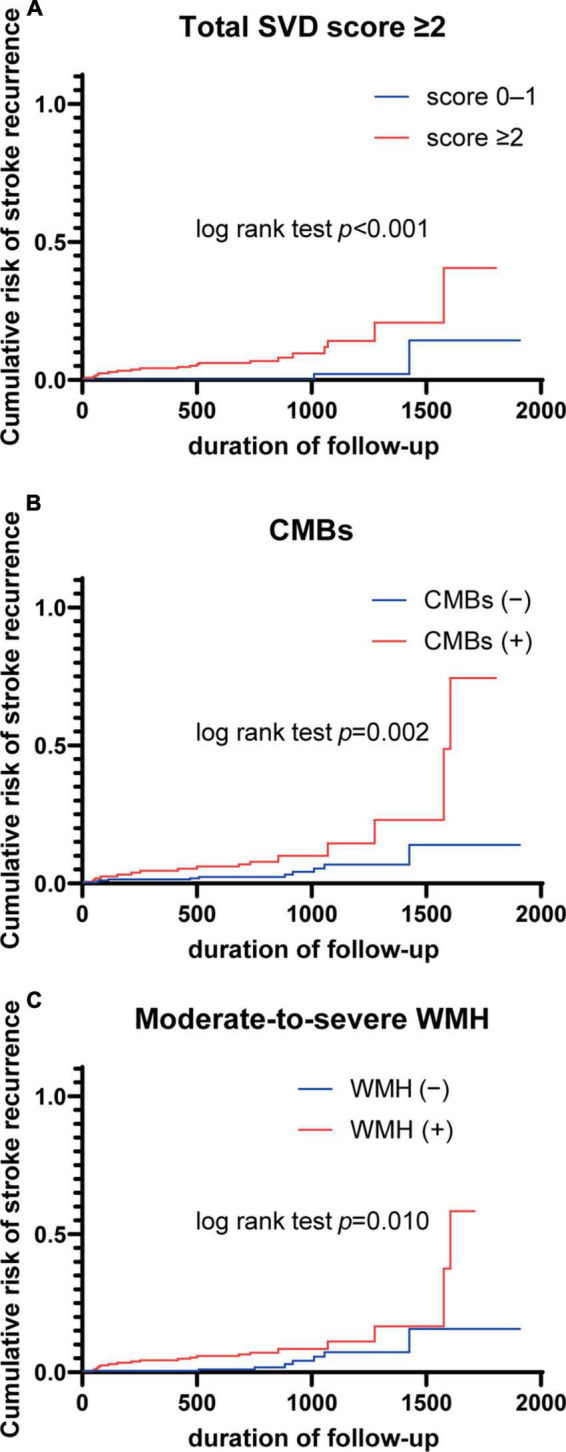
Kaplan–Meier curves for stroke recurrence according to total SVD score and neuroimaging markers. **(A)** Total SVD score ≥ 2; **(B)** CMBs; **(C)** moderate-to-severe WMH. CMBs, cerebral microbleeds; SVD, small vessel disease; WMH, white matter hyperintensity.

Hazard ratios (HRs) for recurrent stroke and all-cause mortality based on total SVD score and individual markers of SVD are shown in Table 4. Multivariate Cox proportional-hazards regression analysis adjusting for age, sex, hypertension, dyslipidemia, diabetes mellitus, atrial fibrillation, and smoking showed that total SVD score ≥ 2 was associated with an increased risk of recurrent stroke events (HR 9.31, 95% CI 2.33–64.23). For individual SVD markers, presence of CMBs and moderate-to-severe WMH were more common in cases with recurrent stroke events (HR 2.90, 95% CI 1.22–6.88; HR 2.81, 95% CI 1.08–7.30, respectively). In a similar analysis for all-cause mortality, neither total SVD score nor any individual neuroimaging marker were associated with increased risk of all-cause mortality. Kaplan–Meier curves for the recurrent stroke event rate of total SVD score ≥ 2, presence of CMBs, and moderate-to-severe WMH are shown in Figure 2.

**TABLE 4:** Prognostic value of the presence of neuroimaging markers.

Presence of neuroimaging marker	Events/ Patients	Unadjusted HR (95%CI)	*p-*value	Age- and sex-adjusted HR (95%CI)	*p-*value	Multivariate^a^ adjusted HR (95%CI)	*p*-value
**Recurrent stroke events**							
Total SVD score ≥ 3	12/147	2.17 (0.94–5.06)	0.068	1.86 (0.78–4.51)	0.160	1.85 (0.74–4.75)	0.188
Total SVD score ≥ 2	21/243	8.12 (2.35–51.17)	< 0.001	8.45 (2.22–56.55)	< 0.001	9.31 (2.33–64.23)	< 0.001
Lacunae	14/158	2.47 (1.13–5.38)	0.023	2.09 (0.95–4.61)	0.069	2.11 (0.92–4.84)	0.076
CMBs	16/158	3.26 (1.50–7.07)	0.003	2.16 (0.85–5.46)	0.104	2.90 (1.22–6.88)	0.016
Moderate-to-severe WMH	20/236	2.85 (1.25–6.54)	0.013	2.69 (1.08–6.67)	0.033	2.81 (1.08–7.30)	0.034
Moderate-to-severe BG-PVS	21/314	2.68 (0.62–11.53)	0.185	2.07 (0.42–10.34)	0.373	2.03 (0.39–10.56)	0.402
**All-cause mortality**							
Total SVD score ≥ 3	34/152	1.40 (0.88–2.20)	0.153	0.95 (0.60–1.49)	0.814	1.01 (0.62–1.61)	0.980
Total SVD score ≥ 2	59/254	2.22 (1.31–4.01)	0.003	1.21 (0.71–2.19)	0.504	1.52 (0.86–2.83)	0.155
Lacunae	42/167	1.24 (0.83–1.84)	0.282	0.97 (0.65–1.44)	0.880	1.04 (0.69–1.56)	0.848
CMBs	41/170	1.23 (0.82–1.82)	0.319	0.88 (0.59–1.31)	0.547	1.03 (0.67–1.55)	0.903
Moderate-to-severe WMH	65/248	1.55 (1.04–2.34)	0.029	0.95 (0.64–1.43)	0.793	1.09 (0.72–1.67)	0.688
Moderate-to-severe BG-PVS	73/327	7.77 (2.44–47.32)	< 0.001	2.41 (0.74–14.83)	0.167	2.09 (0.63–12.96)	0.263

^a^Adjusted for age, sex, hypertension, hyperlipidemia, diabetes, atrial fibrillation, and smoking. BG-PVS, perivascular spaces in basal ganglia; CMBs, cerebral microbleeds; SVD, small vessel disease; WMH, white matter hyperintensities.

## Discussion

The purpose of this longitudinal prospective cohort study was to investigate the association between severity of SVD and long-term prognosis in an IS cohort from a single primary stroke center in Japan. This paper presented baseline data, including the distribution of neuroimaging markers, and clarified that high-burden SVD as determined by total SVD score ≥ 2 was strongly associated with the incidence of recurrent stroke. In addition, the presence of CMBs and moderate-to-severe WMH were associated with a higher incidence of recurrent stroke. The accumulation of SVD biomarkers as determined by total SVD score was found to offer a more reliable predictor of stroke recurrence than individual biomarkers alone, confirming the utility of total SVD score as a reliable visual scale for estimating comprehensive SVD burden. These findings provide important insights into the mechanisms underlying SVD and highlight the complex relationship between SVD and recurrent stroke.

Although prior research has investigated correlations between total SVD score and cognitive decline or mortality (Song et al., 2017; Del Brutto et al., 2021; Pasi et al., 2021), relatively few studies have examined the association between total SVD score and recurrent stroke (Lau et al., 2017; Chen et al., 2021). In a previous population-based study, we reported that total SVD score ≥ 2 was associated with increased risk of cerebro-cardiovascular events (Suzuyama et al., 2020). An earlier prospective cohort study found a correlation between higher total SVD score and incidence of stroke (Lau et al., 2017). Our findings agree with and extend these previous studies, and this was the first investigation to evaluate the effects of various SVD features among Japanese IS patients on multiple clinical outcomes.

Furthermore, our study revealed that individual neuroimaging markers were associated with an increased risk of recurrent stroke events, a conclusion supported by previous research linking SVD to recurrent stroke. For example, a large-scale, pooled analysis of 38 cohorts including 20,322 IS patients found that the presence of CMBs was associated with increased risk of recurrent stroke (Wilson et al., 2019). In addition, a literature review by Rensma et al. demonstrated that individual neuroimaging markers are associated with poor functional outcomes, cognitive decline, and increased mortality following stroke (Rensma et al., 2018). Our data, however, did not support any role for BG-PVS in increasing the risk of recurrent stroke in our IS cohort. We have previously reported BG-PVS as markers of hypertensive angiopathy (Yakushiji et al., 2014), but that significance appears limited, as comparatively few studies have established correlations with long-term prognosis (Rensma et al., 2018). BG-PVS are thought to appear from early in the course of SVD (Staals et al., 2014) and were present in the majority of patients in our stroke cohort. Such results suggest that pathological severity among neuroimaging markers is not equivalent. For example, a previous study raised the scoring cutoff for BG-PVS from > 10 to > 20 (Lau et al., 2017).

Other studies have demonstrated that total SVD score and individual neuroimaging markers correlated with all-cause mortality (Song et al., 2017; Rensma et al., 2018), but our findings appear to contradict this association. Despite higher total SVD score, our study population showed a comparable relative mortality rate when compared to the Korean population (Song et al., 2017). These discrepancies may be attributed to variations in study samples and cohort characteristics, such as age, which can significantly impact SVD and mortality. In addition, disparities in healthcare systems, including differences in post-stroke recovery care, may also play a role. Overall, the present findings imply that the impact of SVD on mortality may be limited in Japan according to underlying characteristics of the population, highlighting the need for further research to better understand the relationship between SVD and mortality.

Our findings have several important clinical implications. First, our results suggest that SVD may play an active role in recurrent stroke events, and that interventions targeting SVD may thus be effective in reducing recurrent stroke events. Second, our findings indicate that SVD may not be a significant contributor to long-term mortality after stroke, highlighting the importance of targeting other risk factors for stroke-related death. Third, our findings suggest that SVD is not only a passive outcome of vascular risk factors, but also a strong active contributor to recurrent stroke events. Although the most appropriate cutoff for total SVD score to indicate an increased risk of recurrent stroke has yet to be established, our findings suggest that low-burden SVD as determined by a total SVD score of 0–1 predicts a decreased risk of recurrent stroke.

This study had several limitations that need to be considered. First, among patients who underwent MRI, heterogeneity could conceivably exist in the assessment of SVD markers given differences in the field strengths of different scanners and variations in the sequence parameters applied. Second, our study was limited by the single-center design, and the results thus may not be generalizable to other populations. Third, we used a single time point assessment of SVD markers, and progression of SVD over time and its impacts on recurrent stroke events and all-cause mortality remain unclear. In addition, our study did not undertake a stratified analysis by subtype of IS, which would have provided valuable insights into associations between SVD markers, recurrent stroke events and all-cause mortality among different subtypes. We hypothesized that the effect is more pronounced in subtypes of small vessel occlusion and large-artery atherosclerosis, but the sample size in this study was inadequate to permit a comprehensive examination of this aspect. Finally, the proportion of patients categorized with small vessel occlusion in this study is lower than that reported in a previous study (Toyoda et al., 2022). This discrepancy may be due to the fact that a higher proportion of patients with cardioembolism, who require reperfusion therapy, are transported to our tertiary-care institution.

In conclusion, this study found that severity of SVD was associated with recurrent stroke. Compared with individual neuroimaging markers, total SVD score may provide more comprehensive information and allow improvements in personalized care for IS patients. Our findings establish a firmer understanding of SVD prognosis that will help guide the future development of strategies against SVD.

## Ethics statement

The studies involving human participants were reviewed and approved by Ethics Committee of Saga University. The patients/participants provided their written informed consent to participate in this study.

## Author contributions

SI, YY, JT, and HH: study conception. SI, YY, JT, ME, SO, MK, KS, MM, CS, TI, YN, HMo, NO, MY, KId, HMi, KIi, KK, and YK: data acquisition. SI, YY, JT, MN, and AO: analysis and interpretation of data. SI, YY, and HH: drafting the manuscript. YY, HI, TA, and HH: supervision of the study. All authors contributed to the article and approved the submitted version.
