# Supplementary material for: Hypertension, cerebral Amyloid, aGe Associated Known neuroimaging markers of cerebral small vessel disease Undertaken with stroke REgistry (HAGAKURE) prospective cohort study: Baseline characteristics and association of cerebral small vessel disease with prognosis in an ischemic stroke cohort
Source: Front Aging Neurosci. 2023 Mar 2;15:1117851. doi: 10.3389/fnagi.2023.1117851 (PMC10018806; doi:10.3389/fnagi.2023.1117851)
Supplement: Supplementary file 1 [file Data_Sheet_1.pdf]

Supplemental materials

Hypertension, cerebral Amyloid, aGe Associated Known neuroimaging markers of cerebral small vessel disease Undertaken with stroke REgistry (HAGAKURE) prospective cohort study: baseline characteristics and association of cerebral small vessel disease with prognosis in an ischemic stroke cohort

Shuhei Ikeda,Yusuke Yakushiji, Jun Tanaka, Masashi Nishihara, Atsushi Ogata, Makoto Eriguchi, Shohei Ono, Masafumi Kosugi, Kohei Suzuyama, Megumi Mizoguchi, Chika Shichijo, Toshihiro Ide, Yukiko Nagaishi, Hodo Mori, Natsuki Ono, Masaaki Yoshikawa, Kiku Ide, Hiromu Minagawa, Kotaro Iida, Kazuhiro Kawamoto, Yoshiko Katsuki, Hiroyuki Irie, Tatsuya Abe, Hideo Hara

Contents

P2. **Supplemental Methods**
P3–7. **Supplemental Table I–V**

Supplemental Methods

Baseline assessments

Using a standardized case record form, data on demographics (e.g., age, sex, educational level, ethnicity), medical history (e.g., personal and family history of cerebro-cardiovascular disease), medication use, physical examination (e.g., weight, height, blood pressure, cholesterol), lifestyle (smoking behavior, alcohol consumption), and pre-stroke functional status as modified Rankin scale score were collected.

Cognitive assessment

Cognitive function tests were performed on lucid patients with four points on 'eye opening' component of Glasgow coma scale to avoid confounding effects by the acute stroke event. Patients completed the comprehensive 20-min neuropsychological test, which spans multiple cognitive domains, including the Japanese version of the Montreal Cognitive Assessment (MoCA-J) and the Mini-Mental State Examination (MMSE). These tests are performed by a dedicated and specially trained research nurse (Y.K.) during hospitalization.

Blood sampling

Fifteen-milliliter fasting blood samples were taken and directly analyzed for serum hematology and biochemistry.

Other physiological or imaging evaluations

All patients underwent 12-lead electrocardiography on admission. Patients were additionally evaluated using 24-h Holter electrocardiography, transesophageal echocardiography, and 4-vessel angiography, as appropriate.

Supplemental Table I. Detailed conditions of MRI modalities (n = 564)

MRI Equipment	Trio Tim (n = 338)	Avanto (n = 143)	MAGNETOM Skyra (n = 32)	Avanto fit (n = 4)	Prisma fit (n = 47)
Manufacturer			SIEMENS		
MFS, tesla	3.0	1.5	3.0	1.5	3.0
T1WI					
TR, ms	500	400	580	480	580
TE, ms	9.2	13.0	10.0	11.0	10.0
FA, degree	70	80	70	70	70
ST, mm	6	6	4	4	4
Gap, mm	1.2	1.2	1.2	1.2	1.2
T2WI					
TR, ms	4500	3800	4500	4000	4500
TE, ms	89.0	93.0	84.0	96.0	84.0
FA, degree	180	180	150	150	150
ST, mm	6	6	4	4	4
Gap, mm	1.2	1.2	1.2	1.2	1.2
FLAIR					
TR, ms	9000	9000	10000	9000	10000
TE, ms	83.0	99.0	115.0	100.0	115.0
TI, ms	2500	2500	2636.8	2500	2640
FA, degree	150	170	150	150	150
ST, mm	6	6	4	4	4
Gap, mm	1.2	1.2	1.2	1.2	1.2
GE-T2*WI					
TR, ms	532–585	650–656	700	723	700
TE, ms	15	25	12.0	20	12.0
FA, degree	15	20	20	20	20
ST, mm	6	6	4	4	4
Gap, mm	1.0–1.2	1.0–1.2	1.2	1.2	1.2
SWI					
TR, ms	27	49	27	49	27
TE, ms	20	50	20.0	40.0	20.0
FA, degree	15	15	15	15	15
ST, mm	3	3	1.5	1.5	1.5
Gap, mm	0	0	0	0	0
DWI					
TR, ms	5800	5200	7930	5000	7930
TE, ms	92.0	91.0	58.0	56.0	49.0
ST, mm	6	6	4	4	4
Gap, mm	1.2	1.2	0	0	0

SWI and T2*WI can be alternatively used.
DWI, diffusion-weighted imaging; FA, flip angle; FLAIR, fluid attenuated inversion recovery; MFS, magnetic field strength; MRI, magnetic resonance imaging; ST, slice thickness; SWI, susceptibility-weighted imaging; T1WI, T1-weighted imaging; T2WI, T2-weighted imaging; T2*WI, T2*-weighted imaging; TE, echo time; TI, inversion time; TR, repetition time.

Supplemental Table II. Criteria for MRI selection

To evaluate SVD (if a patient has multiple MRI data)
I. ≤3 months from admission
II. Having T2*WI or SWI
III. Having T1WI, T2WI, and FLAIR
IV. Evaluated by 3.0 tesla MRI if other requirements are the same
V. As early as possible from symptom onset if other requirements are the same

FLAIR, fluid attenuated inversion recovery; MRI, magnetic resonance imaging; SVD, small vessel disease; SWI, susceptibility-weighted imaging; T1WI, T1-weighted imaging; T2WI, T2-weighted imaging; T2*WI, T2*-weighted imaging.

Supplemental Table III. Definitions for neuroimaging features

**Lacunae**<br>Focal, sharply demarcated lesions >3 mm in diameter showing high intensity on T2-weighted imaging and low intensity on T1-weighted imaging. They are distinguished from perivascular spaces by their larger size, spheroid shape, and surrounding hyperintensity on FLAIR.
**Cerebral microbleeds**<br>Small (<10 mm) areas of signal void with associated blooming seen on T2*-weighted imaging or susceptibility-weighted imaging. They are rated using the Microbleed Anatomic Rating Scale and categorized into "strictly lobar", "strictly deep or infratentorial", or "mixed lobar and deep or infratentorial".
**White matter hyperintensities and periventricular hyperintensities**<br>Signal abnormality of variable size in the white matter and periventricular that shows hyperintensity on T2-weighted and FLAIR imaging. They were assessed with both white matter hyperintensities and periventricular hyperintensities of the Fazekas scale.
**Perivascular spaces in basal ganglia**<br>Small, sharply delineated structures of cerebrospinal fluid (or very similar) signal intensity, measuring <3 mm following the course of perforating or medullary vessels. Perivascular spaces are rated in basal ganglia.

FLAIR, fluid attenuated inversion recovery.

Supplemental Table IV. The details of inter-rater reliability and intra-rater reliability of SVD markers on MRI

	Inter-rater reliability	Intra-rater reliability
	Calculated with comparison between the certified neuroradiologist (M.N) and neurologists (Y.Y. or J.T.) using 40 randomly selected scans	Determined from 20 randomly selected scans scored twice after four weeks
Lacunae	0.59–0.61	0.68–0.83
Any CMBs	0.88–0.94	0.86–1.00
Lobar CMBs	0.81–0.90	0.90–1.00
Deep CMBs	0.68–0.88	0.89–1.00
Infratentorial CMBs	1.0 (both)	1.00–1.00
Moderate-to-severe WMH	0.58–0.75	0.69–0.75
Severe PVH	0.66–0.69	0.80–0.94
Moderate-to-severe BG-PVS	0.63–0.72	0.79–0.83

BG-PVS, perivascular spaces in basal ganglia; CMBs, cerebral microbleeds; MRI, magnetic resonance imaging; PVH, periventricular hyperintensities; WMH, white matter hyperintensities

Supplemental Table V. Baseline laboratory data

	Ischemic stroke (n = 564)
Blood samples	
Total cholesterol, mg/dl	177.7 ± 40.8
LDL cholesterol, mg/dl	109.4 ± 35.2
HDL cholesterol, mg/dl *	51.5 ± 16.0
Triglycerides, mg/dl	96.2 ± 54.4
Glucose, mg/dl †	98.0 (87.8–118.0)
Hemoglobin A1c, %	5.7 (5.4–6.1)
Creatine, mg/dl †	0.8 (0.7–1.1)
eGFR, ml/min/1.73m^2 †	61.5 (48.1–76.8)

Mean ± standard deviation or median (interquartile range)
All data was <5% missing.
* *p* <0.01, † *p* <0.001 *p* values for differences between cohorts in mean and median scores are based on Mann-Whitney U test; *p* >0.05 for others.
eGFR, estimated glomerular filtration rate; HDL, high-density lipoprotein; LDL, low-density lipoprotein
